# Microperimetry and Adaptive Optics Imaging Reveal Localized Functional and Structural Changes in Asymptomatic *RPGR* Mutation Carriers

**DOI:** 10.1167/iovs.64.1.3

**Published:** 2023-01-06

**Authors:** Danial Roshandel, Tina M. Lamey, Jason Charng, Rachael C. Heath Jeffery, Terri L. McLaren, Jennifer A. Thompson, John N. De Roach, Samuel McLenachan, David A. Mackey, Fred K. Chen

**Affiliations:** 1 Centre for Ophthalmology and Visual Science, The University of Western Australia, Nedlands, Western Australia, Australia; 2 Ocular Tissue Engineering Laboratory, Lions Eye Institute, Nedlands, Western Australia, Australia; 3 Australian Inherited Retinal Disease Registry and DNA Bank, Department of Medical Technology and Physics, Sir Charles Gairdner Hospital, Nedlands, Western Australia, Australia; 4 Department of Optometry, School of Allied Health, University of Western Australia, Western Australia, Australia; 5 Centre for Eye Research Australia, Royal Victorian Eye and Ear Hospital, Melbourne, Victoria, Australia; 6 Ophthalmology, Department of Surgery, University of Melbourne, Melbourne, Victoria, Australia

**Keywords:** retinitis pigmentosa, X-linked carrier, retinitis pigmentosa GTPase regulator (RPGR), adaptive optics imaging, microperimetry

## Abstract

**Purpose:**

Female carriers of *RPGR* mutations demonstrate no significant retinal dysfunction or structural change despite a characteristic tapetal-like reflex. In this study, we examined localized changes of pointwise sensitivity (PWS) and cone density (CD) using microperimetry (MP) and adaptive optics (AO) imaging in female carriers of *RPGR* mutations.

**Methods:**

In this cross-sectional case-control study, MP (MAIA, 10-2 test grid) and AO imaging (rtx1) were performed in female carriers of *RPGR* mutations and unrelated age-matched healthy controls. PWS at 68 loci located 1 degree to 9 degrees away from the preferred retinal locus and CD at 12 loci located 1 degree to 3 degrees away from the foveal center were measured. Severity of defect was defined by standard deviation (SD) from age-matched healthy control means: normal (<1 SD from normal average), moderate defect (1–2 SD from normal average), and severe defect (>2 SD from normal average).

**Results:**

Twelve patients from seven unrelated families were enrolled. Seven patients were asymptomatic, 5 of whom had visual acuity 20/20 or better in both eyes. PWS and CD were available in 12 and 8 patients, respectively. Severe PWS and CD defect in at least 1 test location was observed in 10 of 12 patients and 7 of 8 patients, respectively. Among the five asymptomatic patients who had normal visual acuity, severe PWS and CD defects were observed in three of five and four of five patients, respectively.

**Conclusions:**

MP and AO imaging revealed early functional and structural changes in asymptomatic *RPGR* mutation carriers and should be considered in clinical assessment of these patients.

Retinitis pigmentosa GTPase regulator (RPGR) is a major component of the photoreceptor connecting cilium and outer segment and it plays a crucial role in cellular trafficking and disc morphogenesis.^1^ Two main protein isoforms are encoded by the *RPGR* gene (OMIM 312610), located on chromosomal region Xp11.^2^ Of these, one is encoded by a 19-exon transcript and is constitutively expressed (RPGR^Const^). The other is encoded by a transcript that shares coding exons 1 to 14 attached to a partially retained intron 15 (also known as open reading frame 15; *ORF15*) isoform (RPGR^ORF15^), which is highly expressed in photoreceptors.^3^ Mutations in *RPGR* account for approximately 6% of inherited retinal diseases (IRDs), 60% to 90% of X-linked retinitis pigmentosa (XLRP) and 10% to 15% of male subjects with simplex retinitis pigmentosa (RP).^2^^,^^4^^–^^6^ Mutations in *ORF15* are responsible for approximately 70% of *RPGR*-related RPs and 11% of all RPs.^7^ *RPGR*-retinopathy is associated with variable phenotypes including rod–cone dystrophy, cone–rod dystrophy, and cone dystrophy in both affected male and female subjects.^8^^,^^9^ *RPGR*-associated RP (also known as retinitis pigmentosa 3; RP3; OMIM 300029) is characterized by severe early-onset retinal dystrophy in affected male subjects and variable phenotypic expression in female carriers.^10^ Given the frequency of *RPGR* mutations among IRDs, this gene is an important cause of blindness in the working-age population.^11^

Although female carriers of *RPGR* mutations generally present with mild phenotypes, severe retinal dystrophy has been reported in approximately 23% of carriers.^8^^,^^12^ Female carriers with severe or progressive retinal degeneration might be candidates for gene therapy trials.^12^ However, eligibility criteria and trial end points in female carriers might differ from male patients and are yet to be explored. The classic clinical sign of the carrier status is a tapetal-like reflex (TLR) on fundus examination and imaging. However, TLR is not a consistent or a pathognomonic sign of *RPGR* carrier status. In addition, visibility of the TLR varies between different modalities and the standard method for documenting and quantifying the TLR remains to be determined.^13^ Spectral-domain optical coherence tomography (SD-OCT) frequently shows thinning of the outer retinal layers, whereas ellipsoid zone shortening and cystoid macular oedema are uncommon, unlike in other types of RP.^9^^,^^10^^,^^13^ In addition, abnormal retinal function, as detected by electroretinography (ERG) and Goldmann visual field test, has been identified in 80% to 100% of *RPGR* mutation carriers.^9^^,^^10^ However, there are limited data on localized retinal sensitivity and cone mosaic parameters.

Microperimetry (MP) features of *RPGR* carriers have seldom been reported. Using the Nidek MP-1 microperimeter, the 10-2 grid mean sensitivity was lower than healthy controls in five of five female carriers of *RPGR* mutations.^13^ However, localized retinal sensitivity variation was not reported. Furthermore, the MP-1 has a narrower range of stimulus intensity (0–20 dB) than the Macular Integrity Assessment (MAIA) and MP-3 microperimetry (0–35 dB), resulting in ceiling and floor artifacts. The second hyper-reflective band of the outer retina on SD-OCT correlates to the inner segment ellipsoid and has been referred to as the ellipsoid zone, previously known as the inner segment/outer segments (IS/OS) junction.^14^^,^^15^ Ellipsoid zone changes have been extensively studied in different forms of RP.^16^ Adaptive optics (AO) retinal imaging enables cone mosaic analysis at a cellular level which can provide additional structural information in patients with apparently preserved outer retinal layers on SD-OCT. We have previously shown that AO flood-illumination ophthalmoscopy can be used for diagnosing retinal dystrophy in asymptomatic patients and detecting disease progression in early-stage RP with preserved ellipsoid zone.^17^^,^^18^ There are no reports of AO flood-illumination ophthalmoscopy findings in female *RPGR* carriers. In one study using an alternative imaging technique, AO scanning laser ophthalmoscopy (AOSLO) in female *RPGR* carriers (*N* = 4) revealed cone density (CD) of >1 standard deviation (SD) lower than the normal average at 0.5 mm eccentricity, but not at 1.5 mm eccentricity.^19^ Herein, we report localized functional and structural characteristics of female carriers of *RPGR* mutations using the MAIA microperimeter and commercial AO retinal camera, respectively.

## Methods

### Participants

Female carriers of *RPGR* mutations were recruited retrospectively as part of the Western Australian Retinal Degeneration cohort, a prospective observational study. Age-matched healthy controls were recruited retrospectively either from the Western Australian Retinal Degeneration study or from another prospective study (The Distortion Scotoma Assessment Study) with the same imaging protocols as the control group for the MP and AO cohorts. Inclusion criteria for the control group were age 18 to 65 years, normal visual acuity, normal ocular examination, no history of ocular disease or surgery, and no systemic disease or medication with known ocular side effects. The study protocol was approved by the Human Ethics Committee of the Office of Research Enterprise, The University of Western Australia (RA/4/1/7226, RA/4/1/7916, 2021/ET000151, and 2021/ET000895), and Sir Charles Gairdner Hospital Human Research Ethics Committee (approval number 2001-053) and adhered to the tenets of the Declaration of Helsinki. Written informed consent was obtained from the participants prior to inclusion in the study. Diagnosis of *RPGR* carrier phenotype was confirmed by an experienced inherited retinal disease specialist (author F.K.C.) based on family history, clinical examination, and ocular imaging and/or electrophysiology. Genetic diagnosis was established through the Australian Inherited Retinal Disease Registry and DNA Bank (AIRDR).

### Clinical Evaluations and Multimodal Retinal Imaging

Complete ophthalmic examination including best-corrected visual acuity (BCVA), slit-lamp biomicroscopy of the anterior segment, dilated fundus examination, and Goldmann applanation tonometry were performed in all subjects. BCVA was measured using the early treatment diabetic retinopathy study (ETDRS) chart and recorded as ETDRS letter score and converted to Snellen equivalents. Automated refraction and keratometry (Ark1, Auto Refractor/Keratometer; Nidek, Gamagori, Japan) were performed and axial lengths were measured using optical biometry (IOL Master; Carl Zeiss Meditec, Inc., Dublin, CA, USA). Full-field electroretinography (ffERG; RETIport 3.2, Roland Consult, Brandenburg, Germany, or in-house custom built) was performed in accordance with International Society for Clinical Electrophysiology of Vision standards.^20^

Ultra-widefield (UWF) pseudocolor and green-light autofluorescence (AF) imaging were performed by Optos camera (California, Optos plc., Dunfermline, UK). Infrared reflectance (IR) and simultaneous fovea-centered macular SD-OCT (Spectralis, Heidelberg Engineering, Heidelberg, Germany) volume scans were performed. SD-OCT volume scans were obtained from a 30 degrees × 25 degrees area consisting of 61 horizontal B-scans separated by approximately 130 µm between each scan. Fundus autofluorescence (FAF) imaging was performed using an SLO camera (Spectralis HRA2, Heidelberg Engineering) with short-wavelength autofluorescence (SWAF; λ = 488 nm) and near-infrared autofluorescence (NIAF; λ = 887 nm) modalities. UWF AF, IR, SWAF, and NIAF images were graded for the presence or absence of the TLR, and SD-OCT fovea-centered B-scans were graded for ellipsoid zone defect by two trained observers (authors D.R. [unmasked] and R.C.H.J. [masked]) and arbitrated by a masked senior retina specialist (author F.K.C.).

### Microperimetry

**Figure 1.**
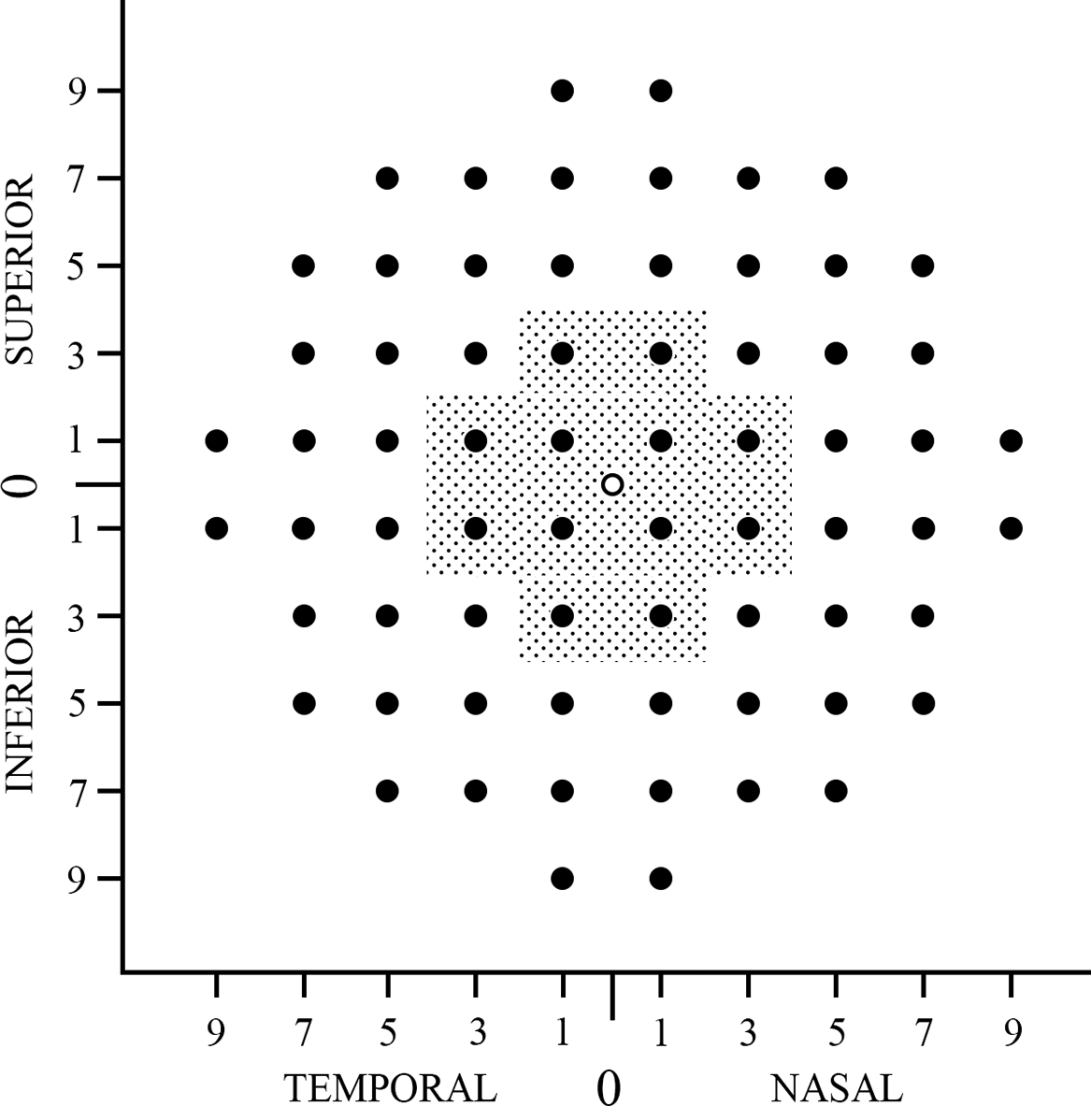
Locations of pointwise sensitivity (PWS) and cone density (CD) measurements. *Solid circles* show the locations of MAIA 10-2 grid (*N* = 68 locations). CD measurement was performed on solid points located within the *dotted area* (*N* = 12). Horizontal and vertical axes show the distance to foveal center (for CD) or preferred retinal locus (for PWS) in degree units. Each location was assigned a horizontal (“T” for temporal and “N” for nasal) and a vertical (“+” superior and “–” inferior) coordinate. For example, 1T, +1 represents 1 degree temporal and 1 degree superior. The *unfilled circle* located at the center represents (0,0) coordinate.

MP was performed by the MAIA microperimeter (CenterVue, Padova, Italy). Exclusion criteria for the MP cohort for patient and control groups were ocular or central nervous system disease with potential impact on visual field, use of medications with known retinal toxicity, fixation inability and spherical equivalent >−8.0 diopters (D). Goldman III achromatic round stimuli with stimulus size of 0.43 degrees diameter and duration of 200 ms were presented with a 4-2 staircase strategy on a dim white background (1.27 cd/m^2^). The range of the stimulus luminance was 0.08 to 317.04 cd/m^2^, which corresponds to 36 to 0 dB sensitivity values. Sensitivity threshold values were classified as normal (>24 dB), relative scotoma (0–24 dB), and absolute scotoma (<0 dB), according to the manufacturer’s recommendations. Loci at which the patient did not respond to the brightest stimulus were assigned as −1. The 10-2 grid (68 test loci) was used to map the pointwise sensitivity (PWS) within the central 20 degrees field (Fig. 1). In addition, fixation parameters including bivariate contour ellipse area (BCEA) 63% and 95%, P1 and P2 (percentage of fixation points that fall within the central 1 degree and 2 degrees, respectively) were recorded. Fixation was considered stable if P1 > 75% and relatively stable if P1 < 75% and P2 > 75%. Patients’ PWS values and fixation parameters were compared with age-matched healthy controls.

### Adaptive Optics Imaging

**Figure 2.**
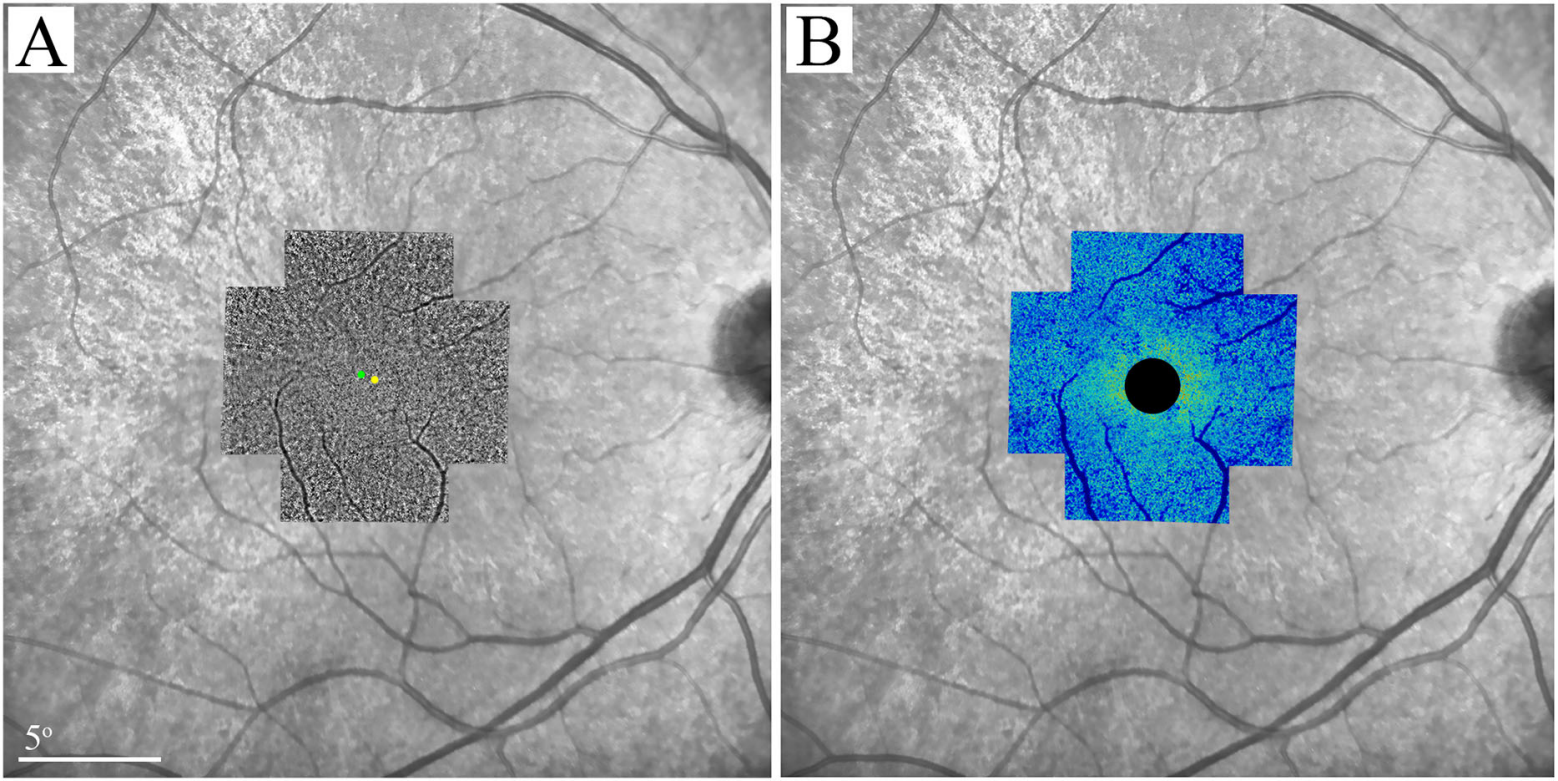
Superimposition of adaptive optics image montage (**A**) and density map (**B**) on 30 degrees infrared reflectance image in patient 6. The *green* and *yellow dots* in panel **A** represent locations of the AO montage center and the foveal pit center, respectively. Due to the limitation of rtx1 camera in visualizing foveal cones, the central 2 degrees (1 degree radius) of the density map has been covered.

Exclusion criteria for AO imaging for patients and controls were BCVA < 20/50, significant cataract or other media opacity, nystagmus, significant cystoid macular edema or epiretinal membrane, spherical equivalent >−6.0 D or cylinder >4 D, history of ocular disease (except carrier status for the patient group) or surgery, and history of using systemic medications with known photoreceptor toxicity. AO imaging was performed using the rtx1 camera (Imagine Eyes, Orsay, France) with the protocol described before.^18^ Briefly, 12 overlapping 4 degrees × 4 degrees image frames centered 2 degrees apart covering the central 6 degrees, focused at the photoreceptor level, were taken. Individual images were stitched together using MosaicJ plugin for ImageJ (Laboratory for Optical and Computational Instrumentation, Madison, WI, USA) and the resultant montage was overlaid on an IR image with marked foveal pit center (Fig. 2A). After careful alignment between the AO montage and the IR image, the foveal pit center was determined and marked on the AO montage and used as the reference point for the localization of the regions of interest.

AO images were analyzed using AODetect version 3.0 (Imagine Eyes, Orsay, France). Cones were detected automatically by the software using an 80 × 80 µm sampling window size and adjusted by an experienced observer (author D.R.). The CD was measured at 12 loci located 1 degree to 3 degrees away from the fovea (see Fig. 1) and angular density values (cells/deg^2^) were reported. Patients’ CD values were compared with those of age-matched healthy controls. In addition, individual image frames were analyzed by an older version of the AODetect software (version 1.0) and stitched together to create a cone density map (Fig. 2B). Because the older version does not provide manual adjustment and used an extra-large sampling window, it was not used for quantitative measurements.

### Genotyping and Pathogenicity Analysis

Genomic DNA was extracted from peripheral blood and stored, as previously described.^21^ Causative familial variants were identified following genetic analysis of a large cohort of affected male subjects clinically diagnosed with XLRP and their family members. DNA from related female carriers in the present study was analyzed by targeted Sanger sequencing of familial variants, alone or in addition to targeted next-generation sequencing of coding and flanking intronic regions of XLRP genes, *RP2* and *RPGR* (including the ORF15 region), or of only the ORF15 region.^22^ Next-generation sequencing was performed by Casey Eye Institute Molecular Diagnostics Laboratory (Portland, OR, USA) or Molecular Vision Laboratory (Hillsboro, OR, USA). Targeted Sanger sequencing was performed by Molecular Vision Laboratory or Australian Genome Research Facility (Perth, Western Australia, Australia). Sequences were aligned to the *RPGR^ORF15^* reference sequence NM_001034853.1, with nucleotide 1 corresponding to the A of the start codon ATG and variants described in accordance with the recommendations of the Human Genome Variation Society.^23^ Variant pathogenicity was assessed as previously described^24^ and interpreted according to the American College of Medical Genetics and Genomics/Association for Molecular Pathology joint guidelines^25^ and associated literature.^26^^–^^29^ In addition to published literature, AIRDR data (unpublished) was used as evidence for pathogenicity assessments.

### Statistical Analysis

Data were recorded in Statistical Package for the Social Sciences (SPSS) version 23 (SPSS/IBM, Inc., Chicago, IL, USA). For each patient, the right eye, or the eye with better BCVA (if >10 ETDRS letters inter-eye difference) was selected for data analysis. Mean and SD of healthy control group were calculated. PWS and CD measurements of individual patients were categorized into normal (within 1 SD of normal average), moderate defect (1–2 SD from normal average), and severe defect (>2 SD from normal average).

## Results

### Mutation Spectrum

Twelve patients from seven unrelated families were enrolled. A causative *RPGR* variant was identified for all pedigrees (Table 1). A total of six different variants – including four deletions and two duplications – were identified. Five were previously reported^7^^,^^22^ and one is novel. All *RPGR* variants identified in this cohort are found within the ORF15 region (*RPGR* transcript, NM_001034853.1) encoding the protein isoform RPGR^ORF15^. Each variant is expected to result in a frameshift and premature termination of the coding sequence and has been classified as either pathogenic or likely pathogenic.

**Table 1.** *RPGR* Mutations Detected in the Cohort

Family	Allele	Protein	Pathogenicity (ACMG)	Reference
1	c.2405_2406del	p.(Glu802Glyfs*32)	Pathogenic	^7^
2, 4	c.2426_2427del	p.(Glu809Glyfs*25)	Pathogenic	^7^
3	c.2045_2046dup	p.(Arg683Valfs*15)	Likely pathogenic	Novel
5	c.2442_2445del	p.(Gly817Lysfs*2)	Pathogenic	^7^
6	c.2635del	p.(Glu879Lysfs*210)	Pathogenic	^19^
7	c.2625dup	p.(Gly876Argfs*203)	Pathogenic	^7^

ACMG, American College of Medical Genetics and Genomics/Association for Molecular Pathology joint guidelines.

### Clinical Characteristics and Multimodal Imaging Findings

**Figure 3.**
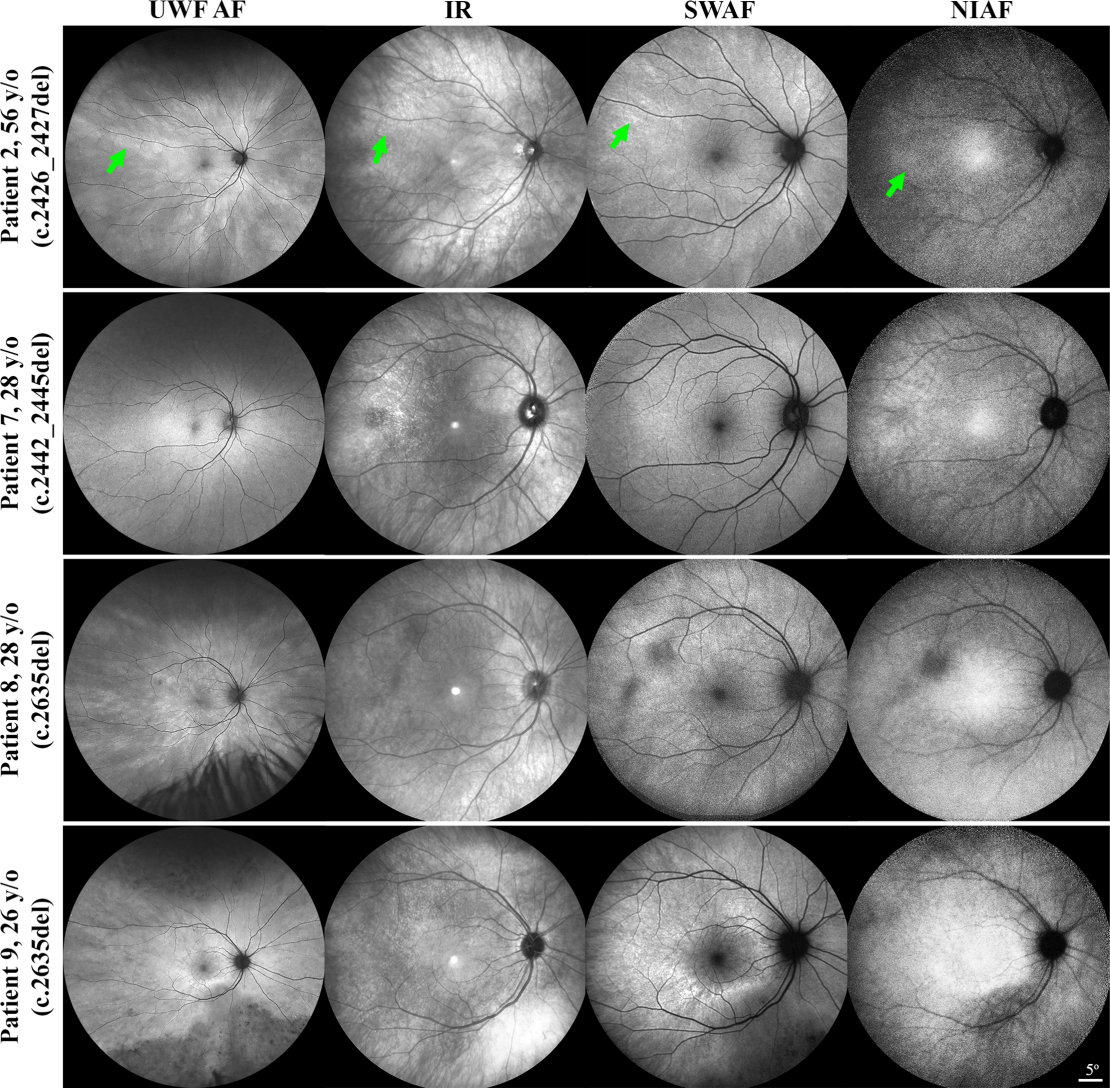
Ultra-widefield autofluorescence (UWF AF), infrared reflectance (IR), short-wavelength AF (SWAF), and near-infrared AF (NIAF) imaging shows variable visualization of the tapetal-like reflex (TLR) in three asymptomatic (patients 2, 7, and 9) and one symptomatic (patient 8) patients. Inferior hypo AF in atrophy areas can be seen in patient 9. *Green arrowheads* show the typical appearance of the TLR on each imaging modality in patient 2. The contrasts of NIAF images have been adjusted to improve the visibility of the reflex. *RPGR* variants are shown in parentheses.

**Figure 4.**
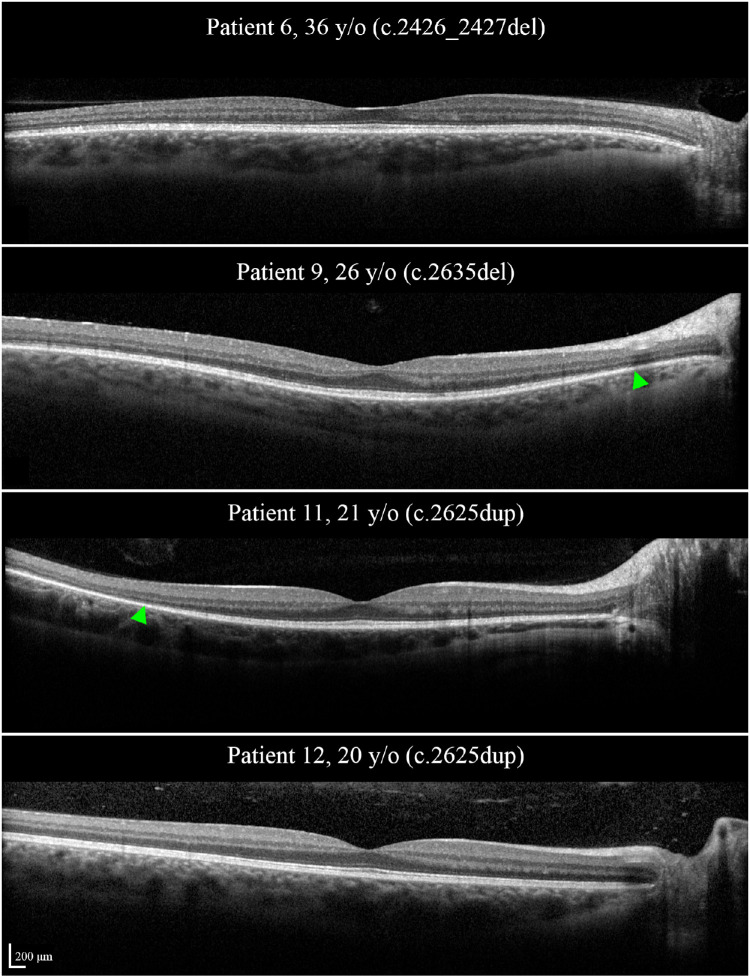
SD-OCT in two asymptomatic (patients 6 and 9) and two symptomatic (patients 11 and 12) patients. Note that ellipsoid zone might be intact or constricted regardless of the symptoms. *Green arrowheads* show the boundaries of the ellipsoid zone. *RPGR* variants are shown in parentheses.

The mean patient age was 36 years (SD [range], 14 [20–63] years). Seven patients were asymptomatic at the time of examination, 5 of whom (aged 25–56 years) had BCVA ≥84 ETDRS letters (Snellen equivalent 20/20) in both eyes. A mild bilateral nuclear sclerosing cataract with no significant media opacification was noted in 3 of 12 patients (Table 2). Fundus examination revealed bilateral TLR in all patients. Patient 1 revealed bilateral asteroid hyalosis, macular and peripapillary atrophy, and pigmentation, and patient 9 revealed bilateral mid-peripheral atrophy and bone-spicule pigmentation. UWF AF and FAF showed bilateral symmetric radiating macular hyperautofluorescence compatible with the TLR in 11 patients and hypo AF in atrophy areas in patients 1 and 9. None of the patients revealed the typical hyperautofluorescent ring on UWF AF or FAF, although an incomplete ring was detected in patient 9 (Fig. 3). SD-OCT was unremarkable in 8 patients and revealed barely detectable ellipsoid zone in patient 1 and peripheral ellipsoid zone defect in the remaining patients (Fig. 4). Fundoscopy, UWF AF, FAF, and SD-OCT were unremarkable, other than the TLR, in all five patients who were asymptomatic and had normal BCVA. Supplementary Table S1 summarizes clinical and multimodal imaging findings in individual patients.

**Table 2.** Demographics and Baseline Clinical Characteristics and *RPGR* Variants Detected in the 12 Patients

				BCVA^*^		SE (D)			
Family	Patient	Age (y)	Onset (y)	RE	LE	RE	LE	Lens	*RPGR* Variant
1	1	63	30^a^	CF	69 (20/40)	−1.50	−0.50	Mild NSC BE	c.2405_2406del
2	2	56	AS	84 (20/20)	85 (20/20)	−2.00	−2.00	Mild NSC BE	c.2426_2427del
3	3	49	NA	60 (20/63)	74 (20/32)	−11.25	−4.50	Mild NSC BE	c.2045_2046dup
	4	33	AS	87 (20/20)	90 (20/16)	+1.00	+1.00	Clear	
	5	32	AS	63 (20/63)	80 (20/25)	−5.00	−3.75	Clear	
4	6	36	AS	90 (20/16)	90 (20/16)	−0.50	−0.75	Clear	c.2426_2427del
5	7	28	AS	89 (20/16)	86 (20/20)	NA	NA	Clear	c.2442_2445del
6	8	28	28^a^	85 (20/20)	80 (20/25)	Plano	−0.75	Clear	c.2635del
	9	26	AS	77 (20/32)	84 (20/20)	−1.50	−1.50	Clear	
	10	25	AS	94 (20/12)	90 (20/16)	+0.25	+0.75	Clear	
7	11	21	20^a^	61 (20/63)	81 (20/25)	NA	NA	Clear	c.2625dup
	12	20	20^a^	65 (20/50)	55 (20/80)	NA	NA	Clear	

AS, asymptomatic; BCVA, best-corrected visual acuity; BE, both eyes; CF, counting fingers; LE, left eye; NA, data not available; NSC, nuclear sclerosing cataract; RE, right eye; SE, spherical equivalent.

a Nyctalopia.

* Early Treatment Diabetic Retinopathy Study (ETDRS) letters. Snellen equivalents are shown in parentheses.

### Microperimetry

**Figure 5.**
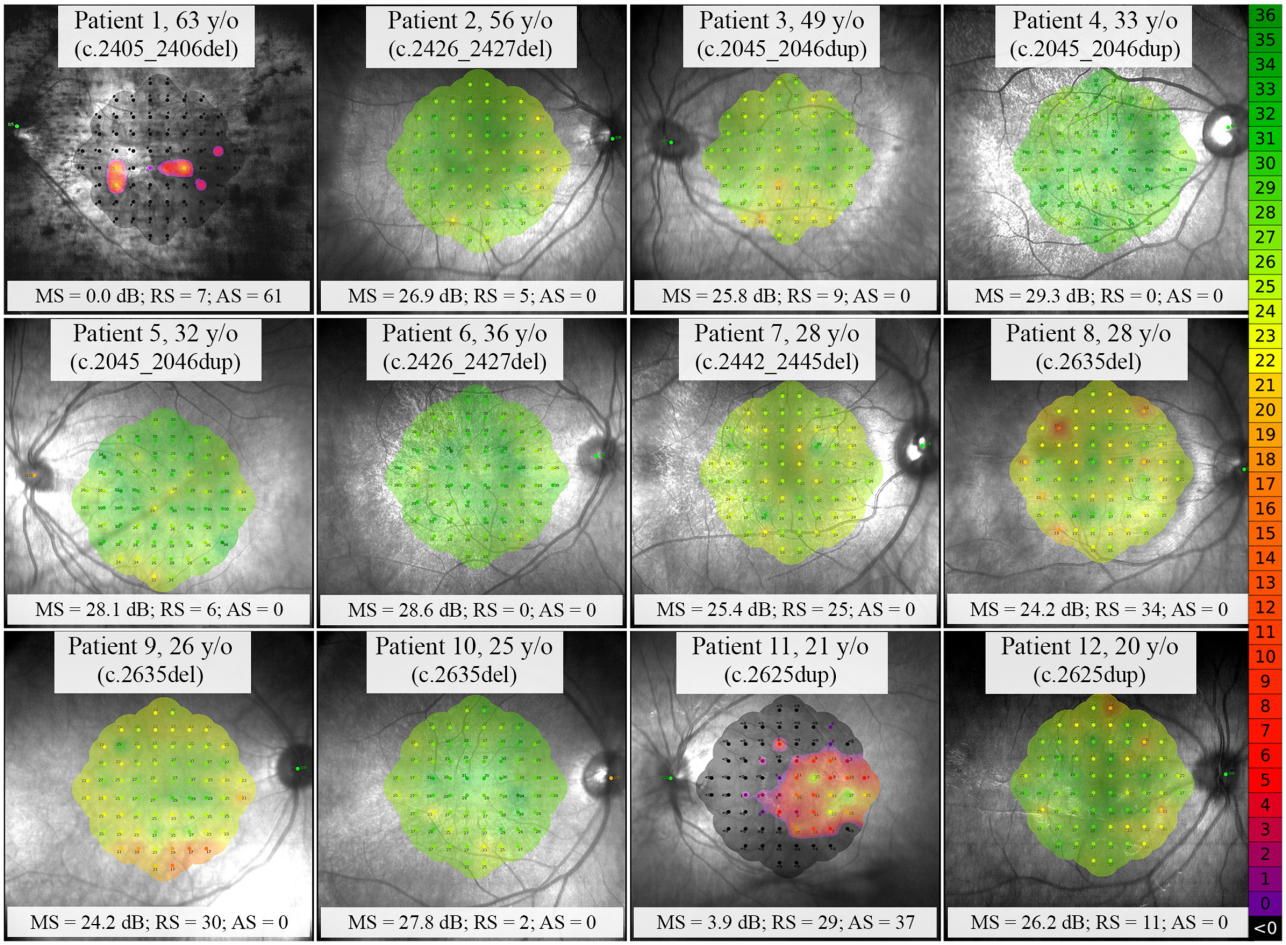
Retinal sensitivity map using the 10-2 MAIA test grid in the 12 patients. Absolute scotoma was observed in only two patients (patients 1 and 11). Pointwise sensitivity (PWS) was normal at all test locations in patients 4 and 6. The remaining 8 patients showed relative scotoma in 2 to 34 of 68 points and no absolute scotoma. MS, mean sensitivity; RS, relative scotoma; AS, absolute scotoma. *RPGR* variants are shown in parentheses.

**Figure 6.**
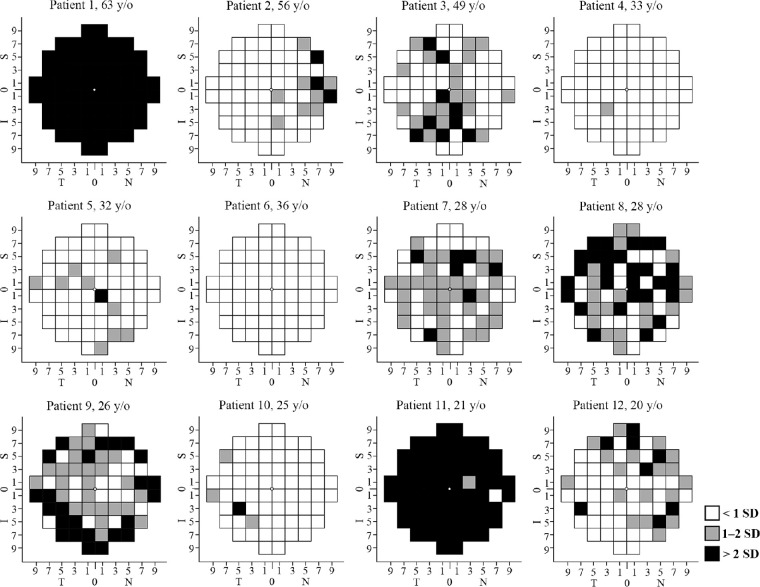
Retinal sensitivity defect map in the 12 patients. A severe pointwise sensitivity (PWS) defect at ≥1 location was detected in 10 patients, including 3 asymptomatic patients with normal visual acuity (patients 2, 7, and 10). Three siblings from family 6 (patients 8, 9, and 10) and 2 siblings from family 7 (patients 11 and 12) showed notable variation in PWS defect. All eyes are transposed to a right eye. N, nasal; T, temporal; S, superior; I, inferior.

Microperimetry was feasible in all patients and PWS data were available in 12 of 12 patients and 25 age-matched healthy controls (Table 3). All patients had stable fixation and BCEA 95% was within 1 SD of the normal average (Supplementary Table S2). Whereas absolute scotoma was observed in only 2 patients (Fig. 5), 10 patients revealed a severe PWS defect in ≥1 test location (Fig. 6). Severe PWS defect was detected in three of five asymptomatic patients with normal BCVA. Of note, MP revealed severe PWS defect at 7 loci and moderate PWS defect at all central loci (within 1 degree) in patient 7 (see Fig. 6) despite lack of symptoms and excellent BCVA. Moreover, PWS showed notable variation among 3 siblings aged 25 to 28 years from family 6 (see Fig. 6, patients 8–10) and 2 siblings aged 20 and 21 years from family 7 (see Fig. 6, patients 11 and 12). PWS at each location and average PWS at each eccentricity in the 12 patients are presented in Supplementary Table S3.

**Table 3.** Characteristics of Patients and Controls Recruited in Adaptive Optics and Microperimetry Cohorts

	MP Cohort			AO Cohort		
	Patient (*N* = 12)	Control (*N* = 25)	*P* Value	Patient (*N* = 8)	Control (*N* = 10)	*P* Value
Sex (M:F)	0:12	19:6	–	0:8	5:5	–
Eye (R:L)	8:4	17:8	–	8:0	10:0	–
Age (y)						
Mean	36	38	0.64	33	36	0.62
SD	14	10		12	13	
Min	20	21		20	21	
Max	63	54		56	54	
SE (D)						
Mean	−1.28	−1.40	0.88	−0.46	−0.66	0.75
SD	1.85	2.08		1.12	1.37	
Min	−4.50	−5.00		−2.00	−3.25	
Max	+1.00	+1.25		+1.00	+1.25	
AL (mm)						
Mean	23.44	24.42	0.05	22.99	23.88	0.06
SD	1.03	0.63		0.71	0.83	
Min	22.21	23.40		22.21	22.40	
Max	25.17	25.40		23.97	25.01	

AL, axial length; AO, adaptive optics; MP, microperimetry; SD, standard deviation; SE, spherical equivalent.

SE was available in 16 controls and 10 patients of MP cohort and all controls and 7 patients of AO cohort. AL was available in 12 controls and 7 patients of MP cohort and all controls and 6 patients of AO cohort. Seven controls were enrolled in both AO and MP cohorts.

### Adaptive Optics Imaging

**Figure 7.**
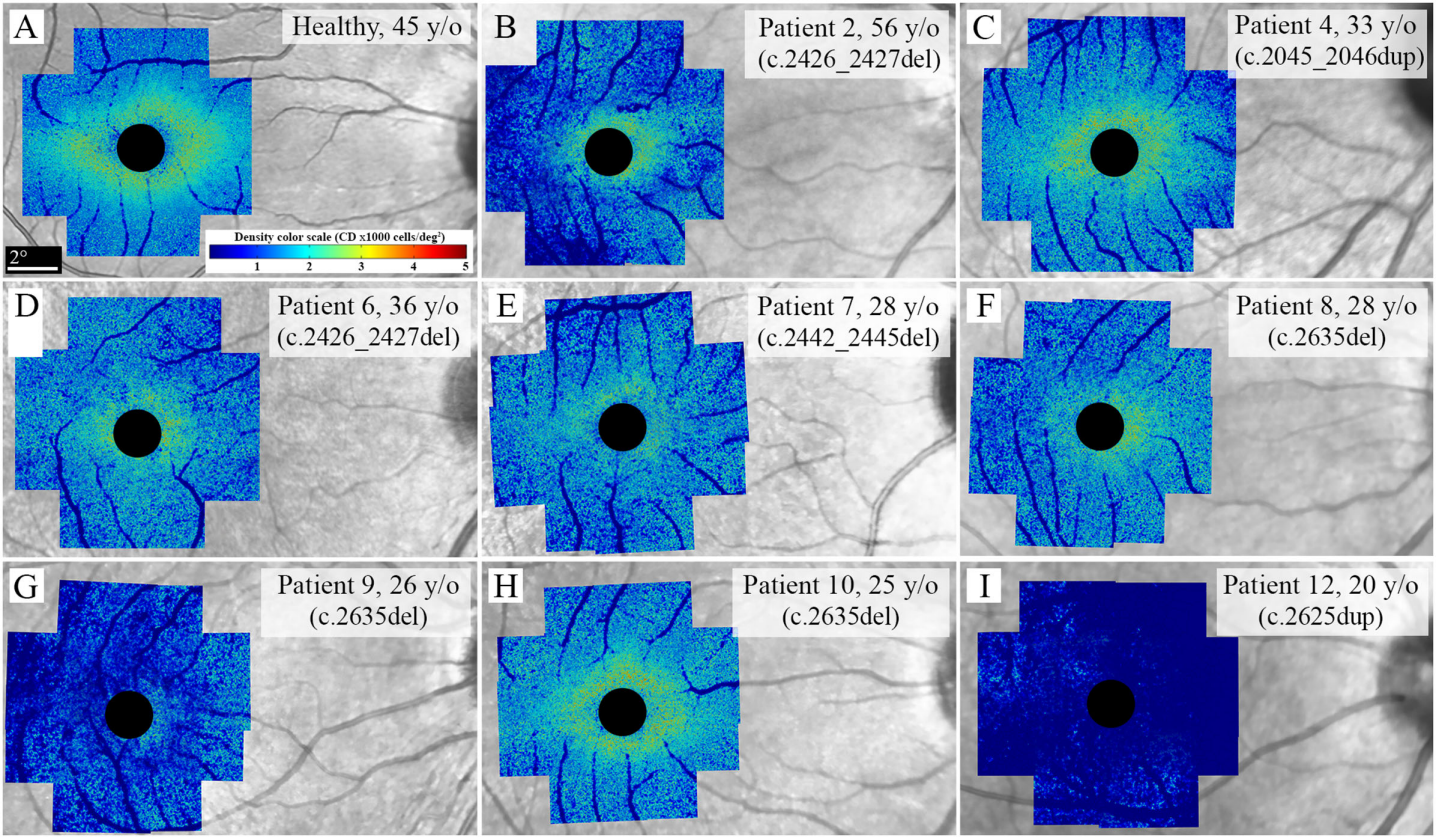
Cone density maps show perifoveal cone loss in patients 4 (**C**) and 10 (**H**), perifoveal and parafoveal cone loss in patients 2 (**B**), 6 (**D**), 7 (**E**), and 8 (**F**) and generalized severe cone loss in patients 9 (**G**) and 12 (**I**). An example of a CD map in a healthy control is presented in **A**. Central 2 degrees (1 degree radius) has been covered due to the limitation of the camera in resolving foveal cones. *RPGR* variants are shown in parentheses.

**Figure 8.**
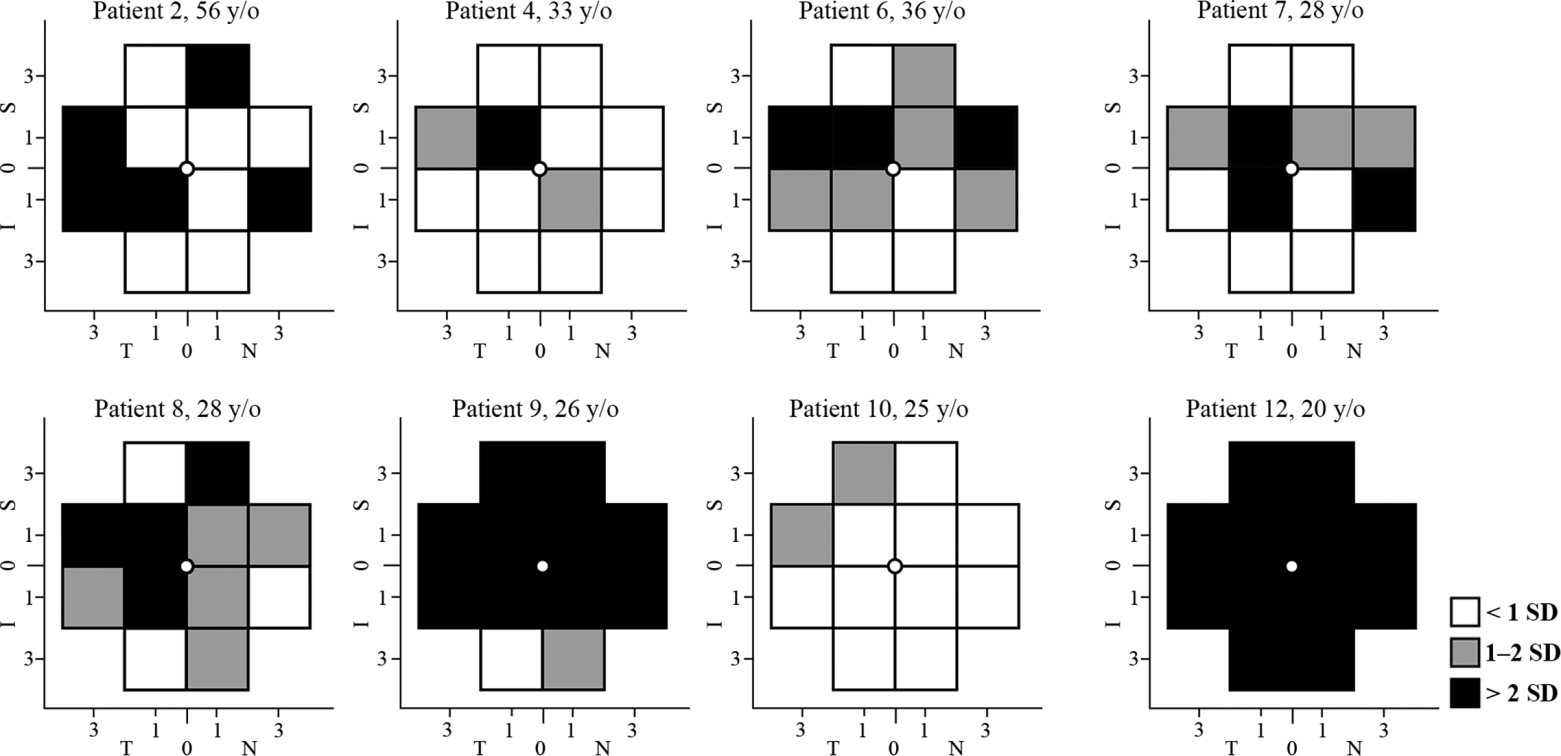
Cone density (CD) defects at various locations in the eight patients. A severe CD defect at ≥1 location was observed in seven patients, including four asymptomatic patients with normal visual acuity (patients 2, 4, 6, and 7). Three siblings from family 6 (patients 8, 9, and 10) showed notable variation in CD defect despite similar age range (25–28 years old). N, nasal; T, temporal; S, superior; I, inferior.

**Figure 9.**
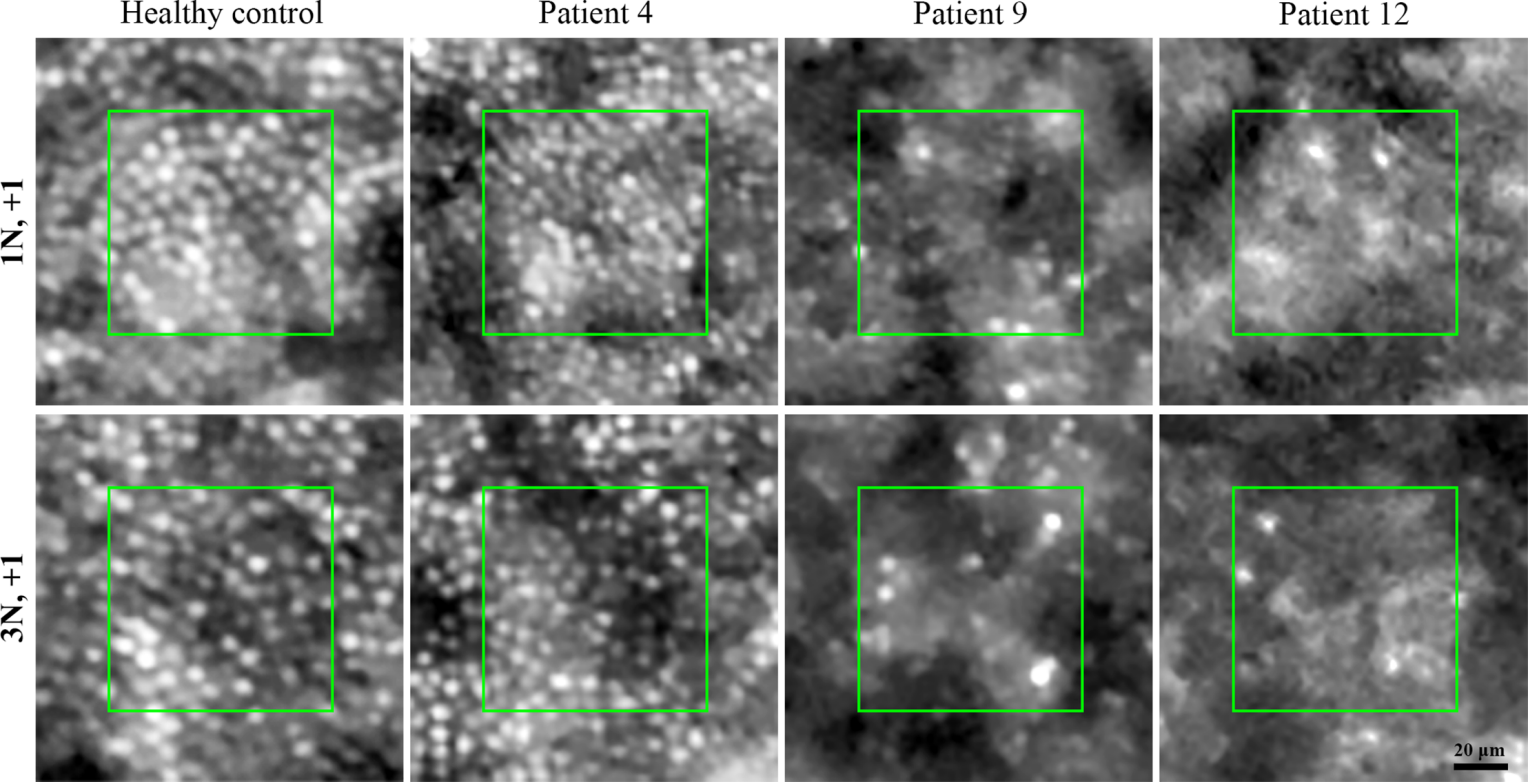
Examples of cone mosaic at two supero-nasal locations in a 33-year-old female healthy control and 3 *RPGR* mutation carriers. Whereas cone mosaic was unremarkable in patient 4, patients 9 and 12 showed severe cone loss at both locations. N, nasal; +, superior.

Flood-illumination AO imaging was performed successfully in all patients. However, four patients were excluded from the CD analysis because of either severe image distortion due to high astigmatism in patients 3 and 5, or undetectable cone mosaic at the regions of interest in patients 1 and 11. Hence, CD data were available in 8 of 12 patients and compared with 10 age-matched healthy controls (see Table 3). CD map revealed decreased parafoveal and/or perifoveal CD in all patients (Fig. 7). Severe CD defect at ≥1 test location was observed in 7 of 8 patients, including 4 of 5 asymptomatic patients with normal BCVA (Fig. 8). In addition, mean CD at 1.4 degrees eccentricity was >2 SD below the normal average in 4 of 8 patients (Supplementary Table S4). Of interest, patient 6, with normal PWS at all locations, revealed severe CD defect at 3 locations and moderate CD defect at 5 locations (see Fig. 8). In addition, the CD defect was more prominent than the PWS defect in patients 2 and 4. Like PWS, there was a notable variation in CD defect among the 3 siblings from family 6 (see Fig. 8, patients 8–10). Examples of cone mosaic in a healthy control and three carriers are shown in Figure 9.

## Discussion

We detected a TLR on multimodal imaging in 11 of 12 patients and an ellipsoid zone defect on SD-OCT in 7 eyes of 5 patients. The TLR has been the most consistent clinical finding in female carriers of *RPGR* and *RP2* mutations, although it may not be detected in all patients.^10^^,^^12^^,^^30^ In addition, despite evidence that supports a relationship between the TLR and retinal structure^31^ and function,^13^^,^^32^ the clinical importance is limited because the TLR was not associated with disease severity^9^ and long-term longitudinal studies showed no change over time.^31^^,^^33^ Similarly, SD-OCT may be unremarkable in *RPGR* mutation carriers. Talib and colleagues showed preservation of all retinal layers in 22 of 47 (47%) patients and varying degrees of outer retinal atrophy in 25 of 47 (53%) patients.^9^ Another study showed intact ellipsoid zone band in the central 30 degrees (9 mm) in 9 of 10 XLRP carriers, with no correlation between the TLR and SD-OCT features.^13^ These findings warrant further investigation to explore techniques that can detect and quantify functional and structural changes in *RPGR* mutation carriers.

Reports on microperimetry findings in *RPGR* carriers are sparse. Genead and colleagues used Spectral OCT/SLO system (version 2.2, OPKO instrumentations/OTI, Miami, FL, USA) that also incorporates a microperimeter to measure PWS at 40 loci within the central 12 degrees in 3 XLRP carriers (unknown genetic diagnosis). They found patches of subnormal PWS over the areas with TLR in all cases, despite normal ellipsoid zone span and retinal layer thicknesses in the posterior pole.^32^ Acton and colleagues observed notable reduction in 10-2 grid mean sensitivity using the MP-1 microperimeter (Nidek Technologies, Padova, Italy) in 9 of 10 XLRP carriers, including 5 of 5 genetically confirmed *RPGR* carriers. There was a relative sensitivity reduction in the inferior macula compared with superior macula. However, this was not correlated with TLR areas, and this asymmetry was also observed in healthy controls.^13^ More recently, Salvetti and colleagues reported generalized bilateral PWS loss on 10-2 MAIA in a 21-year-old female *RPGR* carrier with severe “male pattern” phenotype. The mean sensitivity in the right and left eyes was 4.9 dB and 6.6 dB, respectively, and the scotoma was deeper at peripheral test loci.^34^ In our cohort, although the mean sensitivity showed severe defects only in symptomatic patients, 10 of 12 patients (including 5 asymptomatic) showed severe defects in ≥1 location.

AO imaging showed cone mosaic defects in asymptomatic patients with IRDs who had preserved retinal structure on SD-OCT and FAF.^17^^,^^18^ *RPGR* carrier status is an example of retinal dystrophies associated with unremarkable retinal structure.^9^^,^^13^ Kalitzeos and colleagues reported a significant (29.4%) reduction of cone density in TLR areas compared to areas unaffected by TLR using AOSLO in 7 *RPGR* mutation carriers.^31^ They also found that cone inner segments were larger, cone outer segments were dimmer, and rod outer segments were brighter in TLR areas compared to non-TLR areas. Reflectivity of photoreceptor outer segments did not change over 5 and 10 months in 2 cases.^31^ However, the authors did not report CD values in comparison with healthy controls. Pyo and colleagues reported CD values at 0.5 mm, 1.0 mm, and 1.5 mm from the foveal center in 5 female XLRP carriers (4 genetically confirmed as carriers of *RPGR* mutations) using an AOSLO camera.^19^ All patients in their study had normal visual acuity, a normal ellipsoid zone band on macular SD-OCT, and a visible TLR on near-infrared reflectance and FAF. They reported significant (>1 SD) reduction in CD at 0.5 mm eccentricity in 4 of 5 patients, compared with age-matched healthy controls. CD at 1.5 mm eccentricity was within 1 SD of normal average in all patients.^19^ We found a greater than 2 SD reduction of CD in 7 of 8 patients, in locations that were consistent with Pyo et al., where 0.5 mm and 1.0 mm are approximately equivalent to angular distances of 1.5 degrees and 3.0 degrees, respectively.

We reported a variation in CD and PWS in the 3 siblings of family 6 despite similar age range. Intrafamilial variation of *RPGR* carrier phenotype has been reported frequently^9^ and attributed to the variation in X chromosome inactivation (XCI) pattern.^35^ In addition, it has been proposed that within eye asymmetry in functional and structural involvement in female *RPGR* carriers may indicate variable localized XCI.^36^^,^^37^ Although genomic DNA analysis can identify individual XCI ratios, ocular tissue analysis is required to confirm localized XCI variations. We did not evaluate genotype-phenotype correlation due to the small sample size and the genotype and phenotype heterogeneity that were observed in the studied group. Similar studies on larger samples are required to explore potential correlations between specific mutations and PWS or CD patterns in female *RPGR* carriers.

A major limitation of our study was the lack of ERG in all patients, which could help determine carrier phenotype (i.e. rod–cone dystrophy, cone–rod dystrophy, and cone dystrophy) and its correlation with CD and PWS. In addition, the small sample size, especially for the control group, may have resulted in large SD and consequent underestimation of CD and PWS defects. We did not correlate CD values with PWS due to technical challenges in precise alignment of the two modalities. We have recently shown that there might be notable misalignments among the AO montage center, foveal pit center, and preferred retinal locus,^38^ which limits the accuracy of structure-function correlation between AO imaging, microperimetry, and visual acuity.

## Conclusions

Our findings support a potential role for AO imaging and microperimetry in assessing localized retinal structure and function in carriers of *RPGR* mutations. CD and PWS may help characterize early structural and functional defects in asymptomatic carriers who manifest normal visual acuity and unremarkable SD-OCT. The more prominent CD defect compared with PWS defect in asymptomatic patients suggests that AO imaging may be more useful in early detection of structural damage in these patients. Longitudinal analysis is recommended to assess the role of CD and PWS as potential end points for future natural history studies and clinical trials.

## Supplementary Material

Supplement 1
