# Supplementary material for: Microperimetry and Adaptive Optics Imaging Reveal Localized Functional and Structural Changes in Asymptomatic RPGR Mutation Carriers
Source: Invest Ophthalmol Vis Sci. 2023 Jan 6;64(1):3. doi: 10.1167/iovs.64.1.3 (PMC9836009; doi:10.1167/iovs.64.1.3)
Supplement: Supplement 1 [file iovs-64-1-3_s001.pdf]

Supplementary Materials

Microperimetry and Adaptive Optics Imaging Reveal Localized Functional and Structural Changes in Asymptomatic *RPGR* Mutation Carriers

Danial Roshandel, Tina M. Lamey, Jason Charng, Rachael C. Heath Jeffery, Terri L. McLaren, Jennifer A. Thompson, John N. De Roach, Samuel McLenachan, David A. Mackey, Fred K. Chen

Table S1. Baseline multimodal imaging findings in the 12 patients.

Table S2. Fixation parameters in the 12 patients in comparison with mean and standard deviation in 25 age-matched healthy controls.

Table S3. Pointwise sensitivity (dB) at 68 locations of MAIA 10-2 test grid in the 12 patients compared with mean and standard deviation in 25 age-matched healthy controls.

Table S4. Cone density (CD) at various locations in the 8 patients compared with mean and standard deviation in 10 age-matched healthy controls.

Table S1. Baseline multimodal imaging findings in the 12 patients.

Family	Patient	BSP	TLR				EZ loss
			UWF AF	IR	NIAF	SWAF	
1	1	BE	-	-	-	-	BE
2	2	-	BE	BE	BE	BE	-
3	3	-	BE	BE	BE	BE	RE
	4	-	BE	BE	BE	BE	-
	5	-	BE	BE	BE	BE	-
4	6	-	BE	BE	BE	BE	-
5	7	-	BE	BE	BE	BE	-
6	8	-	BE	BE	BE	BE	-
	9	BE	BE	-	BE	BE	BE
	10	-	BE	-	BE	BE	-
7	11	-	BE	BE	BE	BE	RE
	12	-	BE	-	BE	BE	-

BE = both eyes; BSP = bone spicule pigmentation; EZ = ellipsoid zone; IR = infrared reflectance; NIAF = near-infrared autofluorescence; SWAF = short-wavelength autofluorescence; TLR = tapetal-like reflex; UWF AF = ultra-widefield autofluorescence.

Table S2. Fixation parameters in the 12 patients in comparison with mean and standard deviation in 25 age-matched healthy controls.

	**Control**			**Patient**											
	**Mean**	**-1 SD**	**-2 SD**	**1**	**2**	**3**	**4**	**5**	**6**	**7**	**8**	**9**	**10**	**11**	**12**
**P1 (%)**	89.4	74.7	60.1	97	77	88	85	89	100	81	89	99	100	96	100
**P2 (%)**	96.8	91.4	86.0	100	93	93	91	98	100	92	93	100	100	99	100
**BCEA 63%**	1.5	3.4	5.2	0.6	2.6	3.5	4.8	1.3	0.1	3.3	3.8	0.3	0.1	0.8	0.1
**BCEA 95%**	6.4	15.8	25.2	5.3	7.9	10.5	14.4	3.9	0.4	9.8	11.5	1.0	0.8	2.4	0.4

BCEA 63% and 95% = the area of the bivariate contour ellipse encompassing 63% and 95% of the fixation points; P1 and P2 = percentage of fixation points within central 1° and 2°, respectively; SD = standard deviation.

Table S3. Pointwise sensitivity (dB) at 68 locations of MAIA 10-2 test grid in the 12 patients compared with mean and standard deviation in 25 age-matched healthy controls.

Locus (deg)	Control			Patient											
	**Mean**	**-1 SD**	**-2 SD**	**1**	**2**	**3**	**4**	**5**	**6**	**7**	**8**	**9**	**10**	**11**	**12**
1N, +1	28.7	25.9	23.2	-1	27	25	34	28	28	24	23	27	30	0	27
1T, +1	28.6	26.1	23.6	-1	27	27	28	24	28	26	29	25	31	11	27
1T, -1	28.9	27.3	25.8	6	29	25	30	30	30	26	27	27	29	15	27
1N, -1	29.1	27.3	25.6	0	27	27	30	24	30	26	25	29	28	3	29
3N, +1	28.7	26.8	24.9	-1	27	27	30	30	30	26	27	27	30	-1	27
3N, +3	28.7	26.2	23.8	-1	29	27	28	28	32	30	23	27	30	2	23
1N, +3	28.7	27.3	25.9	-1	29	27	32	28	30	22	23	27	29	-1	29
1T, +3	28.4	25.9	23.4	-1	27	27	30	30	28	26	27	25	29	4	25
3T, +3	28.8	26.2	23.6	-1	27	27	28	26	36	26	23	25	29	9	27
3T, +1	28.3	26.1	24.0	-1	29	27	30	28	30	26	23	27	29	25	29
3T, -1	28.6	26.1	23.7	14	29	29	30	30	30	24	27	27	29	11	29
3T, -3	28.1	26.3	24.4	-1	27	25	26	28	28	26	25	25	27	11	27
1T, -3	27.8	26.0	24.1	-1	27	25	28	26	30	24	23	25	27	3	29
1N, -3	28.0	26.0	24.0	-1	27	21	26	30	30	28	23	23	27	5	29
3N, -3	28.4	26.6	24.8	-1	27	25	32	26	28	26	25	25	28	0	29
3N, -1	28.8	26.6	24.4	-1	27	25	32	30	30	24	23	29	28	2	25
5N, +1	28.5	25.9	23.2	-1	25	27	34	32	32	26	21	25	28	-1	29
5N, +3	28.1	26.3	24.5	-1	27	27	30	30	28	26	27	27	28	-1	25
5N, +5	27.0	24.5	22.1	-1	27	25	30	28	28	24	25	25	30	-1	21
3N, +5	28.0	26.1	24.3	-1	27	25	32	26	30	24	25	25	29	-1	27
1N, +5	27.7	25.9	24.1	-1	29	27	30	30	30	24	27	25	27	8	25
1T, +5	28.2	26.6	25.1	-1	29	25	28	28	30	26	25	25	29	-1	29
3T, +5	27.7	25.7	23.7	-1	27	25	28	30	28	24	23	25	29	-1	25
5T, +5	27.6	26.1	24.5	-1	29	27	28	28	28	24	15	29	27	0	27
5T, +3	28.0	25.5	22.9	-1	29	29	28	28	28	26	23	23	27	13	29
5T, +1	28.2	26.4	24.7	-1	29	29	30	26	30	26	25	25	31	9	29
5T, -1	27.8	26.0	24.3	-1	27	27	30	30	28	26	25	25	29	21	27
5T, -3	27.2	25.2	23.3	4	29	27	30	28	28	26	25	23	23	23	27
5T, -5	27.2	25.2	23.1	-1	27	25	30	26	30	26	25	23	29	7	27
3T, -5	27.4	25.5	23.5	-1	27	23	30	28	28	30	29	23	25	11	27
1T, -5	28.3	26.1	23.8	-1	27	25	28	28	28	26	29	27	27	17	29
1N, -5	27.8	25.9	24.0	-1	25	23	30	28	30	26	27	25	27	-1	25
3N, -5	27.0	25.0	23.0	-1	29	25	28	28	30	26	25	27	29	-1	23
5N, -5	27.4	25.4	23.3	-1	29	27	28	28	28	24	23	23	28	-1	23
5N, -3	27.6	25.7	23.9	15	25	27	32	30	28	26	27	25	30	-1	27
5N, -1	28.6	26.3	24.0	15	29	27	32	30	28	26	27	29	34	1	27
7N, +1	28.0	26.0	23.9	-1	23	27	30	26	28	24	23	23	28	-1	27
7N, +3	27.8	26.0	24.3	-1	27	27	30	28	26	24	23	27	28	-1	25
7N, +5	27.5	25.5	23.6	-1	23	27	30	34	28	24	25	23	28	-1	25
5N, +7	27.4	25.3	23.2	-1	25	25	26	28	28	26	19	23	29	-1	25
3N, +7	27.4	25.7	24.0	-1	27	25	30	30	28	26	23	23	27	-1	27
1N, +7	27.8	25.7	23.7	-1	27	27	28	30	30	26	23	23	27	-1	23
1T, +7	27.8	24.9	22.1	-1	27	27	30	30	30	26	23	23	27	-1	25
3T, +7	27.4	25.4	23.4	-1	27	23	28	30	28	26	23	25	27	-1	23
5T, +7	27.3	25.4	23.5	-1	27	25	26	28	28	24	23	23	27	0	25
7T, +5	27.1	25.2	23.4	-1	27	27	28	26	28	26	23	23	25	-1	27
7T, +3	27.5	26.0	24.4	-1	27	25	28	28	30	28	23	25	27	3	27
7T, +1	27.7	25.7	23.7	4	29	27	28	30	26	24	27	27	27	13	27
7T, -1	28.0	26.4	24.7	-1	29	27	28	30	28	26	27	23	27	27	29
7T, -3	27.3	25.3	23.4	-1	27	25	32	28	30	24	21	23	27	19	23
7T, -5	26.9	24.7	22.5	-1	27	27	30	34	28	24	25	25	27	-1	27
5T, -7	27.4	25.4	23.4	-1	27	23	26	28	28	26	19	21	27	-1	29

3T, -7	27.1	24.8	22.5	-1	23	23	28	26	28	22	23	19	27	-1	27
1T, -7	27.8	26.4	25.0	-1	27	23	32	28	28	26	25	23	27	-1	27
1N, -7	26.5	22.3	18.1	-1	27	25	28	26	28	26	23	19	23	-1	27
3N, -7	27.2	25.3	23.4	-1	27	21	30	24	28	24	23	17	25	-1	27
5N, -7	27.0	24.7	22.3	-1	27	23	28	24	26	24	25	17	27	-1	23
7N, -5	27.1	25.0	22.9	-1	25	27	28	30	28	24	25	23	28	-1	23
7N, -3	27.4	24.7	22.1	-1	23	27	30	26	28	24	23	25	28	-1	21
7N, -1	27.4	25.7	24.0	-1	25	27	28	30	28	26	23	25	28	-1	25
9N, +1	26.9	25.3	23.7	-1	25	27	26	26	26	26	25	23	28	-1	25
1N, +9	26.9	24.5	22.2	-1	27	25	28	28	30	26	23	25	29	-1	19
1T, +9	27.4	25.2	23.0	-1	27	27	32	30	30	26	25	23	27	-1	25
9T, +1	27.4	25.2	23.0	-1	27	27	30	24	30	24	21	23	27	7	25
9T, -1	28.1	25.8	23.6	-1	27	27	28	28	28	26	23	23	25	21	29
1T, -9	27.0	25.0	23.0	-1	27	25	30	26	28	24	23	21	27	-1	25
1N, -9	25.9	22.2	18.5	-1	25	25	28	22	28	26	25	17	25	-1	27
9N, -1	27.4	25.4	23.4	-1	23	25	30	28	30	26	25	21	28	-1	27

SD = standard deviation.

T = temporal; N = nasal; “+” = superior; “-” = inferior.

Table S4. Cone density (CD) at various locations in the 8 patients compared with mean and standard deviation in 10 age-matched healthy controls.

**Locus (deg)**	**Control**			**Patient**							
	**Mean**	**-1 SD**	**-2 SD**	**2**	**4**	**6**	**7**	**8**	**9**	**10**	**12**
1N, +1	1910	1779	1667	2139	2193	1726	1692	1701	1124	2258	420
1T, +1	2022	1901	1781	1973	1718	1741	1719	1511	1211	1932	696
1T, -1	2005	1889	1773	1714	2175	1816	1282	1533	1280	2115	178
1N, -1	2004	1891	1777	1894	1864	1999	1891	1877	1137	1978	791
**Mean**	**1983**	**1861**	**1739**	**1930**	**1988**	**1821**	**1646**	**1656**	**1188**	**2071**	**521**
3N, +1	1614	1463	1312	1572	1809	1301	1316	1455	943	1772	170
1N, +3	1436	1292	1149	781	1556	1187	1348	932	1105	1476	191
1T, +3	1402	1255	1107	1451	1334	1352	1267	1274	923	1165	822
3T, +1	1689	1536	1382	738	1333	1222	1497	958	969	1452	674
3T, -1	1614	1389	1163	836	1609	1329	1490	1192	907	1613	527
1T, -3	1322	1198	1075	1246	1383	1353	1257	1542	1274	1431	297
1N, -3	1355	1203	1051	1325	1476	1398	1216	1177	1143	1231	708
3N, -1	1549	1430	1311	1272	1548	1419	1298	1648	1119	1647	697
**Mean**	**1502**	**1306**	**1111**	**1153**	**1506**	**1320**	**1336**	**1272**	**1048**	**1473**	**511**
**Total mean**	**1657**	**1371**	**1086**	**1412**	**1667**	**1487**	**1439**	**1400**	**1095**	**1673**	**514**

SD = standard deviation.

T = temporal; N = nasal; “+” = superior; “-” = inferior.
